# Entorhinal cortex stellate cell synchronization

**DOI:** 10.1186/1471-2202-15-S1-P93

**Published:** 2014-07-21

**Authors:** Patrick Crotty, Betty K Anderson, Mary Rose Devine, Anna Miettinen

**Affiliations:** 1 Department of Physics and Astronomy, Colgate University, NY 13346, USA

## 

**Figure 1:**
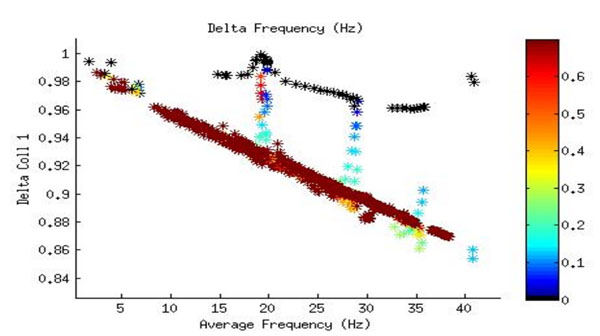
Long-term synchronization of two coupled entorhinal cortex stellate cell neurons with slightly different intrinsic firing frequencies. The mean of their intrinsic frequencies is plotted against a parameter (“Delta Coll 1”) relating the time series of peaks to the eigenvalues of the adjacency matrix, with 1 indicating completely in-phase synchronization and 0 completely out-of-phase. The color bar gives the difference in each intrinsic frequency from the mean (i.e., one-half their difference from each other).

Theta-frequency oscillations (8-12 Hz) of large groups of synaptically coupled cells are commonly seen throughout the mammalian brain. The theta oscillations of entorhinal cortex grid cells and hippocampal place cells have received special attention recently due to their role they apparently play in the encoding of positional information. In a previous study [1], we found that biologically realistic computational models of two or three synaptically coupled entorhinal cortex stellate cells [2] generally synchronize their oscillations much more quickly from an initially random phase configuration if their intrinsic firing frequencies are near the theta range (with the absolute minima generally at about 15-20 Hz) than at significantly either higher or lower ranges. However, our model looked only at identical neurons with the same intrinsic frequencies and lacking any channel noise. Here we present results from further studies in which realistic channel noise and frequency heterogeneity are included. We found that channel noise could either increase or decrease the time for coupled stellate cells to synchronize by a significant amount, depending on which channel the noise was applied to. We also found that the synchronization time appears to be largely independent of the underlying cell parameters that determine the intrinsic frequencies (the persistent sodium and hyperpolarization-activated cation conductances) and depend only on the frequencies themselves, potentially simplifying the process of parameter sweeps. We found evidence that synchronization is favored for a mean firing frequency of 18 Hz even when the individual intrinsic frequencies of the neurons differ by up to about 0.5 Hz (see Figure 1). Finally, we found evidence of a bifurcation in the stability of synchronization states of two coupled identical stellate cells, with in-phase synchronization suddenly switching to out-of-phase synchronization as the synaptic coupling strength is increased, suggesting a complex picture of entorhinal cortex synchronization patterns similar to previous studies of Hodgkin-Huxley neurons [3].
